# Persistent changes in spinal cord gene expression after recovery from inflammatory hyperalgesia: A preliminary study on pain memory

**DOI:** 10.1186/1471-2202-9-32

**Published:** 2008-03-13

**Authors:** Rustam Yukhananov, Igor Kissin

**Affiliations:** 1 Neurogenomic Laboratory Department of Anesthesiology, Perioperative and Pain Medicine, Brigham and Women's Hospital, Boston, MA 02115, USA; 2 Behavioral Laboratory, Pain Research Center, Department of Anesthesiology, Perioperative and Pain Medicine, Brigham and Women's Hospital, Boston, MA 02115, USA; 3 Harvard Medical School, Boston, MA 02115, USA

## Abstract

**Background:**

Previous studies found that rats subjected to carrageenan injection develop hyperalgesia, and despite complete recovery in several days, they continue to have an enhanced hyperalgesic response to a new noxious challenge for more than 28d. The study's aim was to identify candidate genes that have a role in the formation of the long-term hyperalgesia-related imprint in the spinal cord. This objective was undertaken with the understanding that the long-lasting imprint of acute pain in the central nervous system may contribute to the transition of acute pain to chronicity.

**Results:**

To analyze changes in gene expression when carrageenan-induced hyperalgesia has disappeared but propensity for the enhanced hyperalgesic response is still present, we determined the gene expression profile using oligo microarray in the lumbar part of the spinal cord in three groups of rats: 28d after carrageenan injection, 24h after injection (the peak of inflammation), and with no injection (control group). Out of 17,000 annotated genes, 356 were found to be differentially expressed compared with the control group at 28d, and 329 at 24h after carrageenan injection (both groups at p < 0.01). Among differentially expressed genes, 67 (39 in 28d group) were identified as being part of pain-related pathways, altered in different models of pain, or interacting with proteins involved in pain-related pathways. Using gene ontology (GO) classification, we have identified 3 functional classes deserving attention for possible association with pain memory: They are related to cell-to-cell interaction, synaptogenesis, and neurogenesis.

**Conclusion:**

Despite recovery from inflammatory hyperalgesia, persistent changes in spinal cord gene expression may underlie the propensity for the enhanced hyperalgesic response. We suggest that lasting changes in expression of genes involved in the formation of new synapses and neurogenesis may contribute to the transition of acute pain to chronicity.

## Background

Pain that persists well beyond the healing of the injury, including surgical wounds, is a major clinical problem [1]. Multiple mechanisms are likely responsible for persistent postinjury pain; some may be related to the transition of acute pain to chronicity, a concept that is supported by reports of the relationship between the intensity of acute postoperative pain and subsequent development of chronic pain after surgery [2-4]. A long-lasting imprint of acute pain may contribute to its transition to chronic pain. In the rat model, acute pain and hyperalgesia following an inflammation disappeared within a few days, but enhanced sensitivity to a new noxious challenge (inflammation) may persist for a very long time [5,6]. Our previous experiments with repeated injections of carrageenan into the hindpaws demonstrated that, well after recovery (2 to 4 weeks later) from hyperalgesia induced by the initial inflammation, repeated-crossover carrageenan injection causes a distant (in contralateral hindpaw) hyperalgesia that was absent after the initial injection. The acute pain and hyperalgesia following an injection of carrageenan disappears within a few days, but despite recovery the enhanced response to a new noxious challenge may persist for a very long time [5,7]. This phenomenon was viewed as an indication of the long-lasting imprint of acute pain in the CNS [7].

It seems plausible that the transition to chronic pain involved modification of gene expression within the spinal cord, which might be a mechanism of long-term imprinting of pain hypersensitivity. However, the picture of gene expression alterations and their role in control of acute and chronic pain is far from clear [8-10]. Multiple pathways seems to be involved, and their elucidation will require global profile studies of gene expression using different models of acute and chronic pain with and without drug intervention. We have found no studies on changes in gene expression long after recovery from inflammatory hyperalgesia. The aim of this study was to characterize changes in gene expression when animals are long recovered from hyperalgesia but have maintained a propensity for enhanced hyperalgesic response. We hypothesized that following the recovery from inflammatory hyperalgesia there were persistent changes in spinal cord gene expression.

## Results

**Figure 1:**
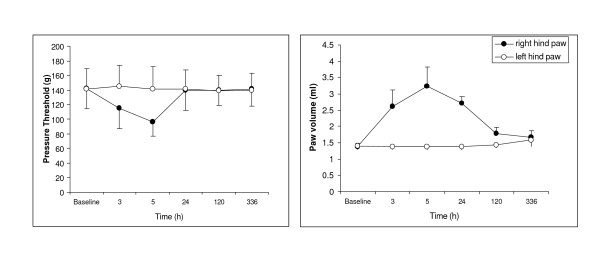
**Effect of carrageenan on noxious pressure threshold and paw volume**. The carrageenan was injected in the plantar surface of the right paw (2%, 0.1 ml). Left Graph. Noxious pressure threshold was measured by Analgesia-Meter (Ugo Basile). Right Graph. Volume measured by a plethysmometer. Data expressed as mean ± SD. The threshold to noxious pressure in the right hindpaw, decreased after carrageenan injection, recovered the next day and was equal to the threshold in the left hindpaw for the whole period of observation (up to 28d).

Carrageenan injection into a rat's hind paw induces inflammation and hyperalgesia (Fig. 1). Inflammation measured as increased paw volume reaches its maximum 3–24h after injection. The differences in paw volume between the injected and contralateral paws are minimal after 5 days and completely disappear in 2 weeks (Fig. 1B). Injection-induced hyperalgesia disappears at 24h (Fig. 1A). However, as was shown in previous experiments, after recovery from hyperalgesia a repeated-crossover injection of carrageenan (in the opposite hindpaw) produced a more exaggerated response, resulting in profound distant hyperalgesia in the originally injected paw even 28d later [7]. In order to distinguish the acute changes induced by inflammation from long-term changes related to pain memory, we collected tissue samples both at 24h following injection, when the inflammation is still significant but there is no hyperalgesia, and at 28d, when as reported previously [7] there is no inflammation but enhanced response to pain stimulus, or "pain memory."

The statistical analysis of gene expression data is still a challenge. There is no standard approach to select differentially expressed genes. Often the combination of p-values and fold differences is used to select "interesting" genes for further analysis [10]. However, to use fold changes for the central nervous system is not efficient, since the changes in gene expression are small and diluted by the large variety of unaffected neurons. In addition, fold changes are not statistically validated metrics and in many cases produce spurious results [11]. In this study, we used the Rosetta Resolver statistical model and based our analysis only on p-value. We compared the number of differentially expressed genes at different p-values. For further analysis, we have used p < 0.01 as a cutoff. It was shown that selecting genes based on p < 0.01 results in more than 80% confirmation rate by real-time PCR [12] and provides a reasonable compromise between stringency and exploratory value of the analysis (Table 1). Using this method we have identified 629 differentially expressed genes in the 28d group and 512 genes in the 24h group out of a total of 31,042 reliably measured transcripts (see Additional file 1). These numbers include both annotated and uncharacterized genes (EST). Further analysis was limited to 356 full-length annotated genes that were differentially expressed compared with the control group at 28d after injection and 329 full-length annotated genes that were differentially expressed compared with the control group at 24h after injection (see Additional file 1). The low variability of our data permitted us to detect highly significant differences with relative changes as low as 10%.

**Table 1:** The number of probe set selected according to p-value.

p-value	28d vs control	24h vs control
All	31042	31042
<0.05	1781	1560
<0.01	629	512
<0.005	441	358
<0.001	177	167

**Figure 2:**
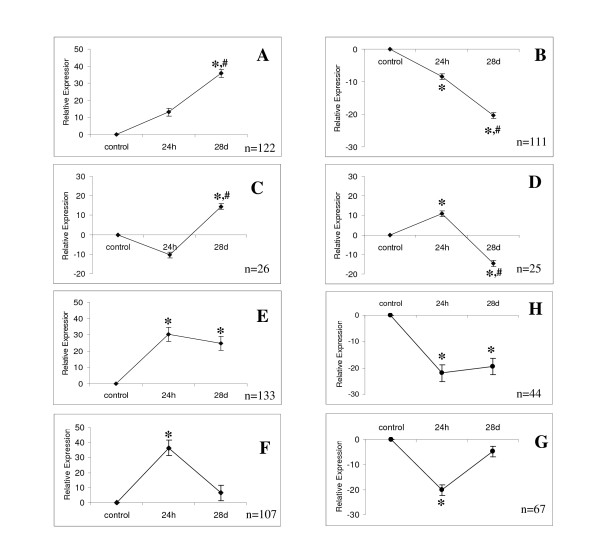
**Clustering of genes differentially expressed at 24h and 28d**. Data presented as mean ± SEM. Genes were classified by SOM; n = number of genes in each cluster. * – p < 0.001 compared to control, #- p < 0.001 compared to 24h group.

In the next step we have characterized the functional groups based on gene ontology (GO) classification using DAVID tools (Table 2 and 3). There are several groups overrepresented only at 24h, when inflammatory response is near its peak (Table 2): protein synthesis/ribosomal protein group and proteins involved in the immune response. Chaperones involved in protein folding were also overrepresented at 24h but with smaller probability. The different groups were overrepresented at 28d (Table 3), such as the genes involved in cell-to-cell interaction, cell proliferation and neurogenesis, morphogenesis, and neuron differentiation–in other words, involved in the formation of new synapses and neurogenesis. DAVID-based GO analysis showed that genes responsible for regulation of apoptosis, phosphorylation, and acetylation were also overrepresented only at 28d group (Table 3). Several functional groups related to regulatory pathways were overrepresented both at 24h and 28d, for example, proteins responsible for ion binding (Table 2 and 3) but it may reflect their overrepresentation in neurons since we have used Affy chip as background for analysis (see Methods). Using SOM, we have grouped the known differentially expressed genes into eight clusters (Fig. 2). Clusters A and B contain the majority of selected genes (n = 233). Cluster A (n = 122) includes genes that increased at 24h after injection and then increased even further at 28d, whereas genes belonging to cluster B (n = 111) changed in the opposite direction: They decreased at 24h and further decreased at 28d. Clusters C and D are similar and include 51 genes that are practically not altered 24h after injection but then slightly increased (cluster C) or slightly decreased (cluster D) at 28d. Genes (n = 177) belonging to clusters E and H increased at 24h (cluster E) or decreased (cluster H) and remained at the same level up to 28d after injection. The last two clusters (G and F) include genes that increased (F) or decreased (G) at 24h and returned to the control level at 28d.

**Table 2:** Ontologic classification of genes differentially expressed at 24h and 28d (B).

**Category**	**Number of sequences**	% of total	p-value
***Protein synthesis/ribosome***			
ribosome	18	5.20%	6.10E-12
protein biosynthesis	19	5.50%	3.60E-10
ribonucleoprotein complex	25	7.30%	1.80E-08
eukaryotic 43S preinitiation complex	7	2.00%	1.60E-05
eukaryotic 48S initiation complex	6	1.70%	6.00E-05
***Protein folding***			
posttranslational protein folding	4	1.20%	2.40E-03
chaperone cofactor dependent protein folding	4	1.20%	1.40E-03
response to unfolded protein	6	1.70%	2.70E-03
***Immune response***			
immunoglobulin domain	16	4.70%	4.90E-05
A-macroglobulin receptor	3	0.90%	1.20E-02
***Regulatory pathway***			
response to stress	*34*	*9.90%*	*5.50E-03*
signal transduction inhibitor	4	1.20%	6.90E-03
cation binding	44	12.80%	3.60E-03
calmodulin binding	6	1.70%	7.50E-03

Functional analysis of differentially expressed transcript employed DAVID tools (see Methods for detailed description). To calculate background, Affychip has been used. Some transcripts represent in more than one category. Cutoff value for category selection p < 0.05.

**Table 3:** Ontologic classification of genes differentially expressed at 28d.

**Category**	**Number of sequences**	% of total	p-value
Cadherin-like	13	3.40%	1.30E-14
cell-cell adhesion	18	4.70%	4.10E-07
***Synapse formation***			
regulation of cell proliferation	20	5.20%	5.70E-04
neurogenesis	15	3.90%	7.60E-03
morphogenesis	34	8.90%	2.70E-03
neuron differentiation	15	3.90%	2.80E-03
***Apoptosis***			
regulation of apoptosis	22	5.80%	1.80E-04
negative regulation of apoptosis	12	3.10%	8.90E-04
***Regulatory pathways***			
calcium ion binding	31	8.10%	2.60E-06
ion binding	62	16.30%	5.50E-04
transmission of nerve impulse	20	5.20%	4.90E-04
actin polymerization and/or depolymerization	7	1.80%	2.40E-04
cytoskeleton organization and biogenesis	18	4.70%	1.70E-03
nerve ensheathment	5	1.30%	4.30E-03
***Enzyme***			
phosphorylation	39	10.20%	9.50E-04
acetylation	14	3.70%	3.20E-03

Functional analysis of differentially expressed transcript employed DAVID tools (see Methods for detailed description). To calculate background, Affychip has been used. Some transcripts represent in more than one category. Cutoff value for category selection p < 0.05.

**Figure 3:**
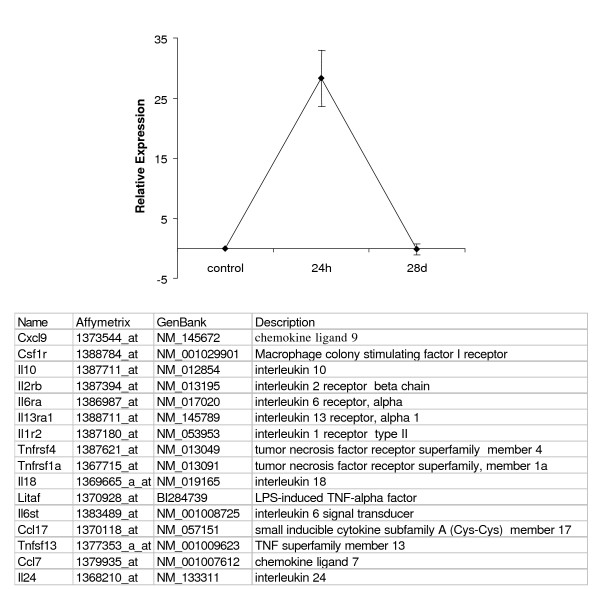
**Profile of gene expression for inflammation-related genes**. The selected inflammation-related genes were clustered with SOM. Data presented as mean ± SEM.

In order to see whether clusters G and F reflect changes in gene expression related to ongoing inflammation, we have selected the group (n = 16) of known mediators of inflammation. They have a similar profile with maximal changes at 24h following injection and practically expressed at the same level as the control group 28d following injection (Fig. 3).

As indirect validation of our result, among differentially expressed genes in both groups we identified (see methods) 67 genes (39 in the 28d group) previously reported to be involved in pain-related pathways, altered in different models of pain, or interacting with protein involved in pain. These genes were divided into several functional groups (Table 4). The first group includes membrane receptors and their ligands. The second contains ion channels, regulators of ion homeostasis, and calcium sensors (e.g., calmodulin) (Table 3). We selected 2 groups that contain signal transduction molecules: the MAPK pathway and proteins involved in cAMP signaling. The fifth group consists of growth factors known to modify pain sensitivity: BDNF [13] and its receptor (NtrkB), neuregulin1, and IGF binding proteins. The sixth group includes critical components of the arachidonic acid (AA) cascade, a known mediator of inflammatory pain.

**Table 4:** Genes differentially expressed 24h or 28d following carrageenan injection and known to be involved in pain.

**GenBank**	**Description**	**Name**	**P-value**	**Cluster**	**Selection criteria**	**References**
	**1. Membrane receptors and their ligands**					

NM_022225	5-hydroxytryptamine (serotonin) receptor 1b	Htr1b	0.0005	A	w	[48]; [49]
NM_012685	vasoactive intestinal peptide receptor 1	Vipr1	0.0017	A	w	[50]
U30290	galanin receptor 1	Galr1	0.0095	B	w	[51]
BI395810	neuropeptide y receptor y1	Npy1r	0.0085	B	w	[52]
NM_017155	adenosine a1 receptor	Adora1	0.0034	E	w	[53]
U28430	5-hydroxytryptamine (serotonin) receptor 3a	Htr3a	0.0002	E	w	[48]
NM_022695	neurotensin receptor 2	Ntsr2	0.0001	E	w	[54]
BF412731	prodynorphin	Pdyn	0.0000	F	w, z	[55]
M11596	calcitonin-related polypeptide, beta	Calcb	0.0000	G	w, z	[56]
BG376217	glutamate receptor, ionotropic, ampa1 (alpha 1)	Gria1	0.0073	H	w	[57]
NM_017011	glutamate receptor, metabotropic 1	Grm1	0.0000	H	w	[37]
NM_012870	neuropeptide y receptor y5	Npy5r	0.0021	H	w, z	[32]; [58]

	**2. Ion channels and regulator of ion homeostasis**					

AI010839	potassium large conductance calcium-activated channel, subfamily m, alpha member 1	Kcnma1	0.0050	A	w	[59]
M34052	potassium voltage gated channel, shaw-related subfamily, member 2	Kcnc2	0.0014	B	w	[59]
AI717104	potassium inwardly-rectifying channel subfamily J member 14	Kcnj14	0.0003	B	w	[59]
NM_032073	potassium voltage-gated channel, subfamily q, member 1	Kcnq1	0.0019	B	w	[59]
AI137995	sodium channel, voltage-gated, type iv, beta	Scn4b	0.0087	B	w	[60]
NM_012618	s100 calcium-binding protein a4	S100a4	0.0066	E	w	[61]
NM_053587	s100 calcium binding protein a9 (calgranulin b)	S100a9	0.0002	F	w, z	[62; 62]
X04280	calbindin 1	Calb1	0.0016	G	w, z	[63]

	**3. cAMP and adenylate cyclase cascade**					

M12672	guanine nucleotide binding protein, alpha inhibiting 2	Gnai2	0.0028	A	y	[64]
U39572	phosphatidylinositol 4-kinase, catalytic, alpha polypeptide	Pik4ca	0.0096	A	w	[65]
NM_053926	phosphatidylinositol-4-phosphate 5-kinase, type ii, alpha	Pip5k2a	0.0067	A	w	[65]
BF410504	g-protein-coupled receptor gpr34	GPR34	0.0000	D	y	[66]
NM_053456	phospholipase c-like 1	Plcl1	0.0000	D	w	[67]
BM386789	regulator of g-protein signaling 1	Rgs1	0.0071	E	w	[68]
BI281632	regulator of g-protein signalling 10	Rgs10	0.0008	F	w	[68]
NM_017142	adenylyl cyclase 8	Adcy8	0.0098	G	y	[35]
NM_134386	g protein-coupled receptor associated sorting protein 1	GASP1	0.0071	G	w, y	[69]
BF565001	phosphodiesterase 4d	Pde4d	0.0053	G	w	[70]
AY043246	regulator of g-protein signaling 2	Rgs2	0.0003	G	w	[38]
NM_017214	regulator of g-protein signaling 4	Rgs4	0.0074	G	w	[38]
NM_080411	g protein-coupled receptor 83	Gpr83	0.0052	H	y	[66]

	**4. MAPK cascade**					

BG664160	mitogen-activated protein kinase kinase kinase kinase 3	Map4k3	0.0027	A	w	[34]
AF092450	mitogen activated protein kinase 8 interacting protein	Mapk8i p	0.0001	A	w	[34]
NM_133545	protein tyrosine phosphatase 2e	Ptpn21	0.0011	A	w	[71]
AI102620	mitogen activated protein kinase kinase kinase 1	Map3k1	0.0027	B	w	[33]
BI290864	mitogen activated protein kinase kinase kinase 3	Map3k3	0.0098	B	w	[33]
AJ271834	protein phosphatase 1b, magnesium dependent, beta isoform	Ppm1b	0.0000	B	w	[72]
BE113127	protein phosphatase 3, catalytic subunit, alpha isoform	Ppp3ca	0.0029	B	w	[72]
AA924620	rab40b, member ras oncogene family	Rab40b	0.0068	B	y	[73]
AI233712	protein phosphatase 1d magnesium-dependent, delta isoform	Ppm1d	0.0011	D	y	[72]
AI104546	protein tyrosine phosphatase 4a3	Ptp4a3	0.0067	E	y	[72]
NM_130403	protein phosphatase 1, regulatory (inhibitor) subunit 14a	Ppp1r1 4a	0.0000	E	y	[72]
AI411122	rab1, member ras oncogene family	Rab1	0.0013	H	y	[73]
BM384841	rab15, member ras onocogene family	Rab15	0.0044	H	y	[73]

	**5. Growth factors and related proteins**					

NM_012513	brain derived neurotrophic factor	BDNF	0.0067	B	w, z	[43] [42]
XM_231739	IGF2 binding protein 3	Igf2bp3	0.0091	D	w	[74]
NM_013104	IGF binding protein 6	Igfbp6	0.0020	E	w	[74]
BE102996	neurotrophic tyrosine kinase, receptor, type 2	Ntrk2	0.0042	E	w	[13]
U02315	neuregulin 1	Nrg1	0.0022	G	w, z	[75]

	**6. Arachidonic acid cascade and mediators of inflammation**					

NM_138547	3-alpha-hydroxysteroid dehydrogenase		0.0055	C	w	[76]
U03389	prostaglandin-endoperoxide synthase 2	cox2	0.0019	D	w	[77]
J04488	prostaglandin d2 synthase	Ptgds	0.0017	E	w	[78]
NM_017020	interleukin 6 receptor	Il6ra	0.0030	F	w	[76]
AA851740	interleukin 6 signal transducer	Il6st	0.0025	F	w	[76]
NM_133551	phospholipase a2, group 4a	Pla2g4a	0.0024	F	w	[79]
AA893169	tissue inhibitor of metalloproteinase 3	Timp3	0.0060	F	w	[9]

	**7. Others**					

NM_012823	annexin a3	Anxa3	0.0026	A	w,z	[14]
BI281979	microtubule-associated proteins 1b	Map1lc 3b	0.0076	A	y, z	[15]
AB011679	tubulin, beta 5	Tubb5	0.0019	A	y	[17]
NM_023974	synaptoporin	Synpr	0.0009	B	y, z	[16]
NM_012551	early growth response 1	Egr1	0.0037	B	w	[29]
BI294912	proteasome subunit, alpha type 3	Psma3	0.0020	D	w	[80]
BI283657	chaperonin subunit 4 (delta)	Cct4	0.0001	F	y	[18]
BG372598	neural cell adhesion molecule 1	Ncam1	0.0045	F	w,z	[81]
NM_012755	fyn proto-oncogene	Fyn	0.0010	E	w	[19]

For cluster description see Result and Figure 3.

Selection criteria:

**w **– involved in pain-related pathways.

**y **– interact with or regulate protein involved in pain perception.

**z**- expression level is altered in different model of pain.

Several genes ("Others", group 7) did not fit in any of these groups. Gene expression of this group (Table 4) has been reported to be altered in different pain models. Annexin 3 level was increased during episodes of migraine [14]; microtubule-associated protein 1B was altered 7 days after chronic constriction injury (CCI) [15]; synaptoporin, a major synaptic vesicle protein in Adelta- and C-fibers and co-localized with calcitonin gene-related peptide (CGRP) in sensory primary afferent neurons, increased after peripheral nerve injury [16].

Several genes were included in group 7 based on indirect evidence of their involvement in pain regulation. Tubulin isoform beta5 interacts with vanilloid receptor (TRPV1) and is probably a downstream effector of TRPV1 activation [17]. Chaperonin subunit 4 (cct4) involved in folding tubulin and other cytosolic proteins is included, since mutation of this protein leads to early onset sensory neuropathy [18]. Fyn proto-oncogene phosphorylates NMDA receptors in response to pain stimuli [19].

**Figure 4:**
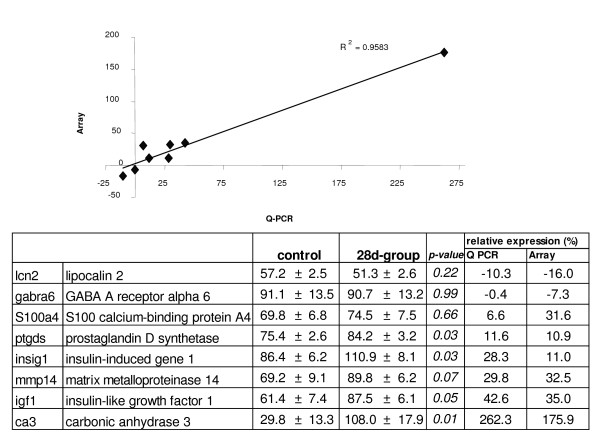
**Comparison of microarray and Q-PCR results**. Equal amounts of total RNA from each sample have been used for PCR amplification. The results were corrected for difference in PCR efficiency based on the dilution curves for each gene. Each gene expression was normalized to reference genes (sep15 and gtpbp4) to correct for possible error in RNA measurement. Data presented as expression relative to reference genes ± SEM. There were 8 control and 9 28d-group samples. p-value is unpaired t-test. Relative expression is presented as percentage of control.

In order to verify the microarray result by independent methods, we randomly selected six of the differentially expressed genes and two non-differentially expressed genes to measure their expression by qPCR (Fig. 4). We found significant (p < 0.001) correlation between expression value obtained using microarray and the real-time PCR results at 28d following carrageenan injection.

## Discussion

The acute pain following a single injection of carrageenan disappears within a few days; however, the enhanced sensitivity to a new noxious challenge may persist for a prolonged time [5-7]. The enhanced sensitivity may contribute to posttraumatic and postsurgical persistent pain. In experiments with repeated carrageenan injections in response to the first injection into a rat's hind paw, the induced hyperalgesia was profound on the side of injection and minimal or absent in the contralateral hind paw. However, repeated injection of carrageenan into the previously noninjected hind paw resulted in pronounced hyperalgesia in the other paw. The difference between distant hyperalgesia after the initial and repeated-crossover injection of carrageenan was used as a measure of the hyperalgesia-related memory. The enhanced hyperalgesia is present even 28 days after the initial injection [7].

In this study we found alterations of gene expression that lasted at least 28d and were quite different from transitory changes observed 24h after injection. The genes altered at 24h include those involved in immune reaction and protein synthesis (Table 2), whereas long-lasting changes observed 28 days after injection indicate different pathways, most notably those responsible for new synapse formation (Table 3).

The observed short-term upregulation of the immune response genes concurs with earlier reports that IL-6 [20,21] and TNF-a (tumor necrosis factor-a) [21] play an important role in inflammatory pain. The upregulation of these genes had previously been associated with the development of neuropathic pain as well [22]. A similar result has been found following nerve injury and spinal cord injury (SCI). SCI induces a robust and significant increase in mRNAs of inflammatory cytokines such as TNF-a, interleukin-1β (IL-1β), and IL-6 at 1, 3, and 24 h post-injury [23-25]. We do not know whether the changes in gene expression are mediated by inflammation, pain, possible injury to the nerve, or a combination thereof.

We observed significant changes in expression of several key enzymes (3-alpha-hydroxysteroid dehydrogenase, phospholipase a2, prostaglandin d2 synthase, and COX2) involved in eicosanoid biosynthesis (Table 4, Arachidonic acid cascade and mediators of inflammation). This cascade is usually initiated by the activation of phospholipase A2 and the release of arachidonic acid (AA) [26]. The AA is subsequently transformed by cyclooxygenase (COX) and lipoxygenase pathways to prostaglandins, thromboxane, and leukotrienes, collectively termed eicosanoids. Eicosanoid production is considerably increased during inflammation and inflammation-induced pain, and COX is the major target for nonsteroidal antiinflammatory drugs (NSAIDs). It is possible that activation of eicosanoids by inflammation/pain causes lasting changes in gene expression. NMDA receptors, which are prominently involved in activity-dependent synaptic plasticity and tonic pain [27], could mediate the upregulation of inflammatory factors. Inflammation-induced NMDAR activation involves phosphorylation of the NR1 and NR2B subunits in the spinal dorsal horn by fyn proto-oncogene [28]. The downstream changes in Egr1, MAPK, and AC expression (see Table 4) can be also induced by activation of the NMDA receptor. As an alternative, activation of the neurotensin receptor can stimulate Egr1 expression and MAP kinase pathways [29].

We do not know whether activation of eicosanoids and MAP kinases is part of one inflammatory/pain pathway or the former is the result of inflammation and the latter is pain-induced. In any case, long-lasting changes in gene expression are unlikely to be induced by ongoing inflammation, since the majority of genes known to be involved directly in the inflammatory response have a profile with maximum changes at 24h and returning to control levels 28d following injection. No detectable changes of inflammatory cytokine genes, including those described above, were found 28d after injection. However, initial inflammatory response may contribute to long-term changes in gene expression and pain memory. A number of genes altered in the 28d group are involved in inflammation-induced potentiation of pain sensitivity. For example, dynorphin [30,31], neuropeptide Y (NPY), and NPY (Y1) receptor [32] are induced in the spinal cord by peripheral inflammation. However, it is unknown whether activation of nociceptors is required.

Based on our expression data, carrageenan injection affected at least two signal transduction pathways, MAPK and cAMP/adenylyl cyclase (AC), known to regulate nociceptive signal perception and transmission. Several isoforms of MAPK are involved in regulation of acute and chronic pain both in neuron and glial cells [33,34]. cAMP mediates many aspects of pain transmission within neuronal cells. In particular, AC isoform 8 (AC8) that couples NMDA receptor activation to cAMP signaling pathways in neurons are important in the development of persistent pain [35]. Activation of MAPK cascade might be mediated by neurotensin receptor via Egr1, which is known to be upregulated in response to persistent inflammatory pain [29] and stimulates Erk1/2 phosphorylation [36]. It is likely that an interaction between the glutamate pathway and cAMP signaling is mediated by modulation of metabotropic glutamate receptor by RGS2 and RGS4 [37,38], which were downregulated 24h after carrageenan injection (Table 4). Based solely on our data, we cannot tell whether the trigger for long-term changes in gene expression is the initial inflammation or pain or their combination. It is also unlikely that all changes in gene expression observed in the 28d group are related to formation and maintenance of nociceptive memory. However, the prominent group (Table 3) of differentially expressed genes controls neuronal connectivity, synaptogenesis, and neurogenesis. It is known that long-term memory and plasticity in CNS depend on formation of new synapses that require synthesis of proteins responsible for cell-to-cell interaction–in particular cadherin-like protein. There is also evidence that formation of new neurons is important for memory formation and plasticity [39].

There is similarity in the development of long-term potentiation (LTP), long-term memory, and pain. Research indicates that central sensitization in the spinal cord has the identical mechanism to LTP [40]. We have found that a number of genes altered at 28d were also altered in the hippocampus during LTP: brain-derived neurotrophic factor (BDNF), early growth response 1 (EGR1), CD9 antigen (CD9), neuropeptide Y receptor Y5 (NPY5R), and neuropeptide Y receptor (NPY1R) [41]. Genes such as BDNF [13,42], NtrkB (BDNF receptor), Egr1 [29], neuropeptide Y receptor, and neuregulin1 are also known to be involved in inflammation-induced pain. For example, BDNF, a known modulator of memory, was found to be altered in rat pups one day after peripheral inflammation induced by injection of Freund's complete adjuvant [43]. We do not believe, however, that pain memory and LTP are identical phenomena but rather share some basic mechanisms. They have different anatomical substrates, and most alterations found in our study were not observed in the hippocampus following memory formation. Based on our data, pain or inflammation or a combination thereof induces dramatic changes in gene expression. Most of these changes in gene expression subside when inflammation disappears, while some of them persist and even increase 28d later. Thus, part of the pain- and inflammation-induced changes in gene expression belong to pathway(s) that remain activated long after inflammation and acute pain disappear. We can only speculate which genes whose expression increased much more at 28d than at 24h belong to these pathways. Additional experiments are needed to narrow down the list of affected pathways. Based on the results of GO analysis, proteins involved in synaptogenesis, cell-cell interaction, and the formation of new neurons are overrepresented among differentially expressed genes at 28d after carrageenan injection. Thus, we propose that pathways related to synapse formation between newly generated neurons are particularly important for "pain memory." The tentative nature of this conclusion depends on the exploratory nature of our microarray data and analysis. The selection of genes is based on a limited number of replicates and multiple comparisons, which implies that a number of genes may be false positives. We confirmed the differential expression for a few selected genes by PCR, but more research is needed to complete verification of the microarray data.

## Conclusion

We have observed long-lasting changes in gene expression following recovery from carrageenan-induced hyperalgesia. On the basis of these data, we propose that pathways related to synapse formation are involved in the formation of pain memory.

## Methods

The study was approved by the Harvard Medical Area Standing Committee on Animals, Harvard Medical School. Experiments were performed on male Sprague-Dawley rats weighing 250–300 g that were housed with a 12-h light-dark cycle and food and water available ad libitum.

Gene expression in the lumbar part of the spinal cord was compared in three groups of rats: 28d after carrageenan injection, 24h after carrageenan injection, and a control group without injection.

Inflammation was induced by injection (26-gauge needle) of 0.1 ml of 2% carrageenan (Sigma Chemical Co., St. Louis, MO) in the plantar surface of the hind paw under halothane (2%) anesthesia. The rat paw volume was measured by a plethysmometer (Paw Volume Meter; Ugo Basile). The general procedure for measuring changes in behavioral responses for microarray experiments was as follows: For several days after arrival, rats were placed in the testing environment, and during the two days preceding the experiment, pressure threshold and hind paw volumes were measured. On the experimental day, basal values were determined twice with a 30-min interval; the average value of two readings was used as a baseline. The rats were assigned randomly (blocked randomization) to one of three groups. Carrageenan was injected into the right hind paw, and the rat was decapitated 24h or 28d after injection. Paw volume was measured on both sides 2h, 4h, 24h, and 28d after each of the carrageenan injections. We assumed that pain-related memory is most likely mediated by altered gene expression both in ipsilateral and contralateral parts of the lumbar enlargement of the spinal cord. In this study we have limited the measurement of gene expression to the ipsilateral portion that is most affected by carrageenan injection.

### RNA isolation and Affymetrix GeneChip processing

The spinal cord was removed under hydraulic pressure with 10-ml syringe with saline [44], and the lumbar enlargement was dissected into ipsi- and contralateral parts and frozen. Tissues were kept at -80°C until RNA isolation.

Total RNA was isolated by quickly homogenizing the tissue at high speed in Trizol (Invitrogen, Carlsbad, CA) according to the manufacturer's instructions. The concentration of total RNA was measured by UV spectrophotometry, and RNA quality was confirmed by electrophoresis in 1% agarose gel and Agilent bioanalyzer (Agilent Technologies, Inc, Santa Clara CA). Only those samples that had a 260/280 ratio more than 1.9 and had no signs of degradation based on agarose electrophoresis were used for analysis. The RNA samples were stored at -80°C.

Gene expression analyses were performed with the Affymetrix Rat Genome 230 v.2 chip (Affymetrix, Santa Clara, CA), which contains ~31,000 probe sets including ~17,000 annotated rat genes. For each chip, RNA from the spinal cord of 3 rats was pooled to minimize interindividual variability. Pooled spinal cord RNA from the control, 24h, and 28d groups was hybridized in triplicate to the Affymetrix 230 rat microarray, so that 9 animals were used per treatment group. The labeling, hybridization, and scanning were done through the NIH Neuroscience Microarray Consortium at Affymetrix GeneChip Resource, W. M. Keck Facility, Yale University School of Medicine, according to Affymetrix protocol using recommended equipment. The DNA chips were scanned by confocal scanner. Digitized image data were processed the MAS software (version 5.0) from Affymetrix. The expression data reported in this paper have been deposited in the National Center for Biotechnology Information Gene Omnibus database.

### Microarray Data Analysis

The resulting microarray data that passed quality control tests were imported into the Rosetta Resolver system for gene expression data analysis (Rosetta Biosoftware, Kirkland, WA). While it was imported, the microarray data were subjected to background correction, intrachip normalization, and the Rosetta Resolver system Affymetrix GeneChip error model. The intensity profiles that are generated were used to build ratios with Rosetta Resolver Ratio Builder. Briefly, the first step in data analysis includes validation of each probe set based on empirical error distribution and removal of the probe pairs that are beyond three standard deviations from the mean. Probe-level data were used to increase robustness by calculating the averages. The expression values for individual profiles were combined using an error-weighted average in a transformed space, and the error of the result was adjusted correspondingly. All profiles belonging to one single experiment are normalized together. We have used global normalization based on weighted average. The Ratio Builder was used calculate ratio probabilities for the differential expression among the control, 24h, and 28d groups. More detailed information on the Rosetta Resolver system Affymetix GeneChip error model, error-weighted statistical model, and ratio building error model can be found at Rosetta Resolver web site and in publications of Hughes et al. [45,46]. As a result of analysis, genes were assigned p-values reflecting differences between the control and injected groups. The p-values were used to select genes for further clustering and functional classification. Thus, the analysis of data was based on the combination of two procedures: Resolver error model and self-organizing map (SOM) clustering complemented by functional classification using DAVID [47]. We have used Affy chip background since more specific list of gene related to spinal cord expression is not available. SOM was applied by the weighted pair-group method with centered average and Pearson correlation implemented in the program xCluster (kindly provided by Gavin Sherlock, Stanford University). The differentially expressed genes were clustered according to their expression in 24h and 28d groups. Statistical differences within cluster were evaluated with one way ANOVA followed by Scheffe test. At the next step, the same genes differentially expressed at 28d and 24h were functionally classified by gene ontology. We also clustered genes known to be involved in inflammation using SOM. In order to organize selected genes according to the intracellular pathways involved, we used a context-based information search and graphical tools (GeneSphere, Malden, MA).

The selection of genes known to be involved in pain perception (Table 3) was based on gene ontology and a context-based information search of PubMed-indexed publications using the following criteria: a. protein involved in pain-related pathways; b. gene expression altered in different pain model; c. interaction with protein involved in pain (Table 4).

### RT-PCR Verification

Quantitative RT-PCR was used to verify the microarray results for six randomly selected differentially expressed genes and two genes that were not differentially expressed on the gene array (negative control). RNA was extracted from each animal as described above and cDNA was synthesized using superscript reverse transcriptase (Invitrogen). PCR amplification used sybr green gene mix from Biorad (Hercules, CA). Cycling parameters were 95°C for 10 min followed by 40 cycles of 95°C for 30 sec, Ta °C for 40 sec, and 72°C for 90 sec. Primers sequences are summarized in Table 5. Amplification of each gene was expressed as the amplification cycle at which its PCR product was first detected (threshold cycle, CT). All samples were run at the same plate. The relative quantification of the target gene expression was performed according to the comparative CT method (ΔΔCT method). For each run, the mean expression levels of target genes were normalized to the mean expression levels of two endogenous reference genes (sep15 and gtpbp4). Serial dilution of standard cDNA was used to correct for difference in amplification efficiency. The "standard" cDNA for the entire study was generated from a tissue homogenate, which was obtained by pooling cDNA from all control rats. PCR buffer and RNA were used as a negative control. The resulting data were analyzed with unpaired t-test. The Pearson correlation coefficient was used to compare fold changes on gene arrays with those found on RT-PCR.

**Table 5:** Sequence of primers for PCR verification.

**Gene**	**Forward Primer**	**Reverse Primer**
gtpbp	CAG CCA AGG ACT TTA TTG ACC	GTC GCA CGT AAT CTT TAG CAA C
sep15.	GAG AAG CTG GAA CGC ATA TAA G	GGG CTA CTT CAG CTT CGA AT
ca3	CAC TGG AAC CCG AAG TAT AAC A	CGC CTT TCT CCC GTC CTA
gabra6	CAA GCC CCC GGT AGC AAA G	TGA CCG GCG TTG ATG GTA AGA T
Igf1	AGC CAA GAC AGA TAG CCA TAC A	AAG CAG AGT GCC AGG TAG AAG
Insig1	CGC TCT TCC CGG ACG AG	CCA CCA CAA GCC CAA GGA TAG G
lcn2	ACT TCA AAG TCA CCC TGT ACG G	ATC CCA GTC AGC CAC GCT CAC
mmp14	TCA GGG CAG TGG ACA GCG AAT A	AGC ACC GGC AGA ACG AC
ptgds	CGG GAG AAG AAA GAG CTA CTG T	GGG CTG TTG TAG GTG TAC TGT C
s100a4	GGC CCT GGA TGT AAT AGT GTC	GCC CTC AAA GAA TTC ATT GCA C

The primers were designed using Oligo software (Molecular Biology Insights, Inc, Cascade, CO)

## Competing interests

The author(s) declare that they have no competing interests.

## Authors' contributions

RY- design, data collection and analysis of microarray data, manuscript preparation IK – design, interpretation of data, manuscript preparation

## Supplementary Material

Additional file 1 The list of differentially expressed genes. The list of genes differentially expressed with p < 0.01 at 24h and 28d following injection of carrageenan with a brief description and relative expression value. Click here for file
